# The Role of Vimentin 3 in Ameloblastomas: A Novel Tumor Biomarker

**DOI:** 10.1111/odi.15396

**Published:** 2025-06-02

**Authors:** Sibel Elif Gultekin, Melanie von Brandenstein, Emre Baris, Ipek Atak Secen, Leyla Arslan Bozdag, Heike Goebel

**Affiliations:** ^1^ Department of Oral Pathology Faculty of Dentistry, Gazi University Ankara Turkey; ^2^ Clinic and Polyclinic for Urology Faculty of Medicine and University Hospital Cologne, University of Cologne Cologne Germany; ^3^ Institute of Pathology, Faculty of Medicine and University Hospital Cologne, University of Cologne Cologne Germany

**Keywords:** ameloblastoma, odontogenic tumor, vimentin 3

## Abstract

**Introduction:**

Ameloblastoma (ABL), a common odontogenic tumor in the maxillofacial region, presents primarily as unicystic (U‐ABL) and conventional (C‐ABL) variants. Despite shared epithelial features, their distinct biological behaviors may stem from interactions between connective tissue and epithelial cells. Vimentin 3 (VIM3), a truncated variant of Vimentin‐Full Length (VIMFL), exhibits unique biological properties. This study is the first to investigate VIM3 expression in ABLs.

**Methods:**

Formalin‐fixed paraffin‐embedded (FFPE) samples of C‐ABL (*n* = 30), U‐ABL (*n* = 30), and dental follicles (DF, *n* = 30) were analyzed. Immunohistochemical evaluation of VIM3, VIMFL, WNT5a, and MTCO1 was performed, alongside qRT‐PCR for VIM3, VIMFL, WNT5a, ROR2, and miR‐498.

**Results:**

VIM3 expression was significantly higher in C‐ABL (*p* < 0.0001) compared to U‐ABL and DFs. VIMFL was absent in the epithelial components of all cases. C‐ABL showed significantly higher WNT5a (*p* < 0.0001) and MTCO1 (*p* = 0.0327) expression. qRT‐PCR revealed significant differences in VIM3 and miR‐498 levels between U‐ABL and DFs (*p* < 0.0001). No significant differences were found for WNT5a, VIMFL, or ROR2 (*p* > 0.05).

**Conclusion:**

This study identifies VIM3 expression in ABLs, distinct from VIMFL, suggesting its potential as a biomarker. Additionally, mitochondrial dysfunction may play a role in ABL tumorigenesis.

## 1 Introduction

Odontogenic tumors include various odontogenic lesions, consisting of benign and malignant neoplasms and hamartomas, each demonstrating distinct biological behaviors (Barnes 2005). The variety of odontogenic tumors, their unique clinical and histopathological attributes, and the rarity of some of these lesions can pose challenges in the diagnosis and treatment within the field of oral pathology.

Ameloblastoma (ABL) is an odontogenic tumor originating from the odontogenic epithelium and is one of the most common odontogenic tumors within the maxillofacial region (Effiom et al. 2018). ABL has a benign histologic character although displaying locally aggressive behavior, often accompanied by a high tendency for recurrence. In the latest classification by the World Health Organization (WHO) in 2022, ameloblastomas are categorized into various subtypes, including conventional (C‐ABL), unicystic (U‐ABL), extraosseous/peripheral, adenoid, and metastatic ameloblastoma (Soluk‐Tekkesin and Wright 2022). Despite their histopathological beingness, each of these variants demonstrates distinct biological behaviors. Among these, the most encountered variants are U‐ABL and C‐ABL. C‐ABLs are classified into six histological subtypes: follicular, desmoplastic, basal cell, plexiform, acanthomatous, and granular cell. On the other hand, U‐ABLs have three subtypes based on the location of the ameloblastomatous epithelium: luminal, intraluminal, and mural (Soluk‐Tekkesin and Wright 2022). U‐ABLs are characterized by a more indolent biological behavior, except for the mural type, often behave as odontogenic cysts, and are typically treated with complete surgical excision (Barış et al. 2015). On the other hand, C‐ABLs exhibit notably aggressive biological behavior. If not adequately removed during the initial surgery, there is a significant risk of local recurrence. The management of C‐ABLs typically involves resection, which can result in substantial facial deformity and associated morbidity and psychological problems for the patients (Mendenhall et al. 2007). The histopathological differentiation between these two lesions, which exhibit significant differences in biological behavior, prognosis, and treatment approaches, can be quite challenging in incisional biopsies. In the literature, there is currently no biomarker available that can determine the prognosis of ABLs and, particularly, differentiate their histological subtypes in small biopsies.

Vimentin 3 (VIM3) protein is a C‐terminal truncated variant of Vimentin, which was discovered by a research group in J. Craig Venter Institute in 2007, and further investigated by von Brandenstein et al. (2015). The Vimentin protein is a crucial component of the cytoskeleton and belongs to the type III intermediate filament family. During embryonic development and wound healing, the expression of vimentin is mostly seen in interstitial cells and migratory epithelial cells (Paulin et al. 2022). Nevertheless, VIM3 exhibits distinct biological functions compared to Vimentin as a result of its distinctive C‐terminal truncation. Intron‐8 is where the truncation takes place, resulting in the insertion of eight extra amino acids. Additionally, von Brandenstein et al. (2018) found that the miR‐498 molecule is also implicated in this process. Since VIM3's discovery, it has been studied in many benign and malignant aggressive tumors, including renal cell carcinoma, oncocytoma (von Brandenstein et al. 2021), and prostate cancer (Köditz et al. 2021a, 2021b). This protein is the only known histological marker used to differentiate chromophobe eosinophilic variant renal carcinoma from oncocytoma (von Brandenstein et al. 2021). Oncocytoma is a tumor composed of oncocytic cells, which is an epithelial cell characterized by an excessive number of mitochondria. Under normal conditions, Vimentin is an essential filament for maintaining cellular structure, whereas VIM3 lacks the terminal portion, resulting in missing tetramers. Consequently, an increased number of organelles move about in the cytoplasm, and a greater quantity of mitochondria can be seen in the cell. Hence, it is hypothesized that there may be a correlation between VIM3 expression and mitochondrial functions (von Brandenstein et al. 2015).

Mitochondrial structure and function constitute the basis of cellular energy metabolism and thus are vital determinants of cell biology in health and disease (Wallace and Fan 2009). It is well known that mitochondrial dysfunction is closely linked to the development of tumors. WNT family proteins are recognized for their crucial roles in mitochondrial quality control and the regulation of energy metabolism (Anastas and Moon 2013). WNT5a, a member of the WNT signaling protein family, consists of 19 hydrophobic, cysteine‐rich glycoproteins observed in humans (Kumawat and Gosens 2016). WNT5a typically activates the non‐canonical WNT/Ca2+ and WNT/planar cell polarity signaling pathways. Additionally, it can either inhibit or activate the canonical WNT/β‐catenin signaling pathway, thereby influencing its function (Anastas and Moon 2013; Kumawat and Gosens 2016). WNT5a has been implicated in the development and progression of several tumor types, including oral cavity tumors, with high WNT5a expression reported in oral squamous cell carcinoma (Prgomet et al. 2015, 2017) and ameloblastoma (Sukarawan et al. 2010). Mitochondrial cytochrome c oxidase 1 (MTCO1), a member of the cytochrome c family, functions as a terminal complex of the mitochondrial respiratory electron transport chain and plays a crucial role in cellular energy production and apoptosis (Eskuri et al. 2021). Although relatively few studies have investigated MTCO1, its expression has been shown to vary across different tumor types (Eskuri et al. 2021; Lin et al. 2016).

**FIGURE 1:**
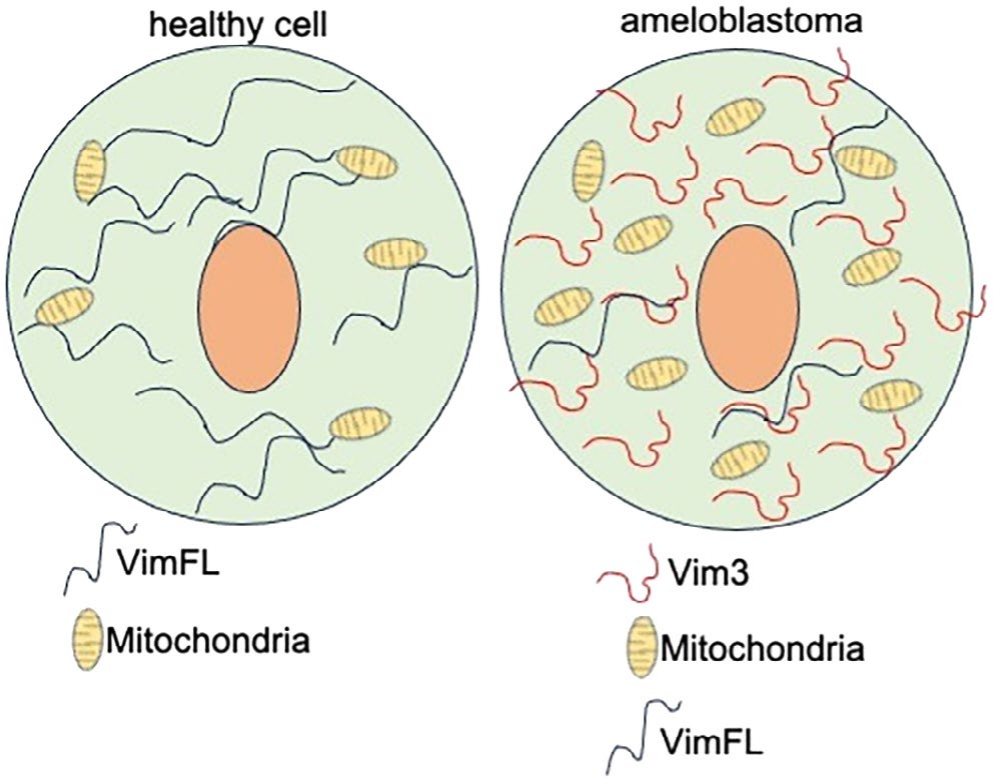
Altered vimentin isoform expression and its impact on mitochondrial dynamics in ameloblastoma cells.

In healthy cells, the number of mitochondria is tightly regulated and varies depending on cell type and metabolic demands, with full‐length Vimentin (VIMFL) being the dominant variant. Since VIM3 exhibits different biological functions compared to VIMFL, VIM3 expression in ABLs is anticipated to be distinct from VIMFL. It was hypothesized that VIM3 truncation would contribute to the aggressive nature of ABL cells by leading to remodeling of the cytoskeleton that facilitates the number of mitochondria, promotes high cellular activity, and increases metabolic capacity (Figure 1).

To date, there are no studies in the literature evaluating VIM3 expression in odontogenic tumors; our study is the first to reveal the VIM3 profile in ABLs. The primary aim of our research is to determine the expression profile of VIM3 in odontogenic tumors, particularly in U‐ABL and C‐ABL. Additionally, another aim of our study is to understand the relationship between VIM3 and mitochondrial functions and to examine the differences between these two lesions.

## 2 Materials‐Methods

### 2.1 Patients

The study was conducted using formalin‐fixed paraffin‐embedded (FFPE) tissue samples obtained from the archives of the Department of Oral Pathology, encompassing a total of 90 previously diagnosed cases. Among the C‐ABL cases (*n* = 30), the follicular type was selected as the most common histologic subtype, while the luminal and intraluminal types were chosen for the U‐ABL cases (*n* = 30). The control group consisted of dental follicles (*n* = 30). All cases localized to the posterior region of the mandible. Ethical approval for the study was granted by the Clinical Research Ethics Committee of Gazi University Faculty of Dentistry (E‐210712825‐050.99‐153,070).

All hematoxylin–eosin stained (H&E) slides from the selected cases were re‐evaluated according to the World Health Organization's latest classification of odontogenic tumors, and the diagnoses were confirmed. For inclusion in the study, FFPEs were selected based on the availability of sufficient tissue for immunohistochemical and molecular analyses. All demographic, radiographic, and macroscopic data of the cases were recorded.

### 2.2 Immunohistochemistry

In each selected sample, areas representing the lesions were marked on the H&E slides. Tissue cores, each with a diameter of 1.2 mm, were extracted using a tissue microarray (TMA) instrument (Beecher Instruments MTA‐1, Estigen OÜ, Estonia) and embedded into empty recipient paraffin blocks. Subsequently, 4 μm sections were cut from the TMA blocks and transferred onto adhesive‐coated slides (Instrumedics Inc., Hackensack, NJ, USA) for immunohistochemistry. The staining procedures were performed manually.

The slides were dried overnight at 37°C, deparaffinized by incubation for 2 × 5 min in xylene, followed by 2 × 3 min 96% ethanol, and 5 min 95% ethanol. To reduce nonspecific background staining due to endogenous peroxidase activity, the slides were incubated in 0.3% hydrogen peroxide in methanol for 20 min at room temperature and then rinsed with tap water. Subsequently, they were pretreated in citrate solution (pH:6) in microwave. After washing with tap water, they were transferred to phosphate‐buffered saline (PBS). The UltraVision Plus Detection System (Thermo Fisher Scientific, USA) used according to the manufacturers' protocol; the slides were incubated in UV Block for 5 min at room temperature to reduce background staining. After washing with PBS, the sections were additionally incubated with 10% bovine serum albumin (BSA). The primary antibodies, VIM3 (provided by Brandenstein/Fries, patent number EP 13160876.2–1405), VIMFL, WNT5a, and MTCO1 were diluted in PBS plus 1% BSA solution and incubated for 60 min at room temperature. The information on the antibodies is provided in Table 1. The anti‐polyvalent was used to detect specific antibodies according to the manufacturers' protocol. For visualization, 3,3′‐diaminobenzidine tetrahydrochloride (DAB) (Thermo Fisher Scientific, USA) was applied. Afterwards, the slides were counterstained with hematoxylin, coverslipped, and mounted.

**TABLE 1:** Antibodies.

Antibody	Clone	Host	Brand	Positive control tissue (human)	Dilution
VIM3	V3	Rabbit polyclonal	EZBiolab, Carmel, ABD	Appendix	1:500
VIMFL	V9	Mouse monoclonal	SantaCruz, Heidelberg, Almanya	Gingiva	1:100
WNT5a	—	Mouse monoclonal	SantaCruz, Heidelberg, Almanya	Placenta	1:1000
MTCO1	1D6E1A	Mouse monoclonal	Abcam, Chambridge, İngiltere	Kidney	1:100

#### 2.2.1 Immunohistochemical Evaluation

The data were assessed independently by three researchers (SEG, EB, and HG) in a blinded manner using a light microscope at ×200 magnification in five randomly selected fields. The expression patterns of VIM3, VIMFL, WNT5a, and MTCO1 were identified in both epithelial and mesenchymal components in all samples. A semi‐quantitative H‐score was calculated based on the staining intensity and pattern (Avilés‐Salas et al. 2017).

### 2.3 Molecular Analyses

The expressions of VIM3, VIMFL, and related microRNA (miR‐498), WNT5a, and its coreceptor ROR2 were detected by using RT‐qPCR, subsequent RNA purification, and cDNA analysis of the FFPE tissues (RNeasy FFPE kit‐Qiagen, Hilden, Germany). For molecular studies, two 15 μ thick slices were extracted from paraffin‐embedded tissue samples and placed into pre‐sterilized Eppendorf tubes. The stages of RNA isolation, RNA quantification, cDNA extraction, and qPCR were conducted in that order. The investigation utilized Vimentin, Vimentin 3, and miR‐498 primers, which Dr. von Brandenstein supplied.

#### 2.3.1 RNA And miRNA Isolation

The RNeasy FFPE kit (Qiagen, Hilden, Germany) was utilized to isolate RNA from FFPE samples, following the instructions provided by the manufacturer. The measurement of RNA was conducted using NanoDrop technology from Thermo Scientific in Oberhausen, Germany. The miR was extracted from cells using the miRNeasy Kit (Qiagen, Hilden, Germany) following the instructions provided by the manufacturer.

#### 2.3.2 cDNA Synthesis

250 ng of RNA was used to produce cDNA using random primers QuantiTect Reverse Transcription Kit (Qiagen, Hilden, Germany) and SuperScript III reverse transcriptase following the manufacturer's instructions (Invitrogen, Darmstadt, Germany). RT‐PCR was conducted according to the methods outlined in earlier studies (von Brandenstein et al. 2015).

#### 2.3.3 qRT‐PCR

A volume of 1 μL of complementary DNA (synthesized from 250 nanogrammes of ribonucleic acid) will be utilized for real‐time polymerase chain reaction analysis. β‐actin was quantitatively analyzed. Every sample was standardized using β‐actin as a reference gene. During the study of miRs, 5 s rRNA was utilized as a normalization factor instead of β‐actin. The ΔΔCT technique was employed for computation, following the guidelines provided in manual 2 (PE Applied Biosystems, Forster City, USA). Cells that did not undergo polymerase chain reaction (PCR) were utilized as a control.

### 2.4 Statistical Analysis

All statistical analyses were performed using GraphPad Prism 5 (GraphPad Software, La Jolla, CA, USA). All experiments in patients with C‐ABL, U‐ABL, and DF were repeated three times. Data on the differences in IHC, mRNA, and miRNA expressions between C‐ABL, U‐ABL, and DF were analyzed using the Kruskal‐Wallis test. The relationship between the two independent numerical variables was analyzed using Spearman's rho correlation coefficient. Data are expressed as *p < 0.05, **p < 0.01, ***p < 0.001. All *p* values were two‐sided, and a *p* value of < 0.05 was considered statistically significant.

## 3 Results

### 3.1 Patients

The mean age of patients with C‐ABL was 35.13 years, with a gender distribution of 18 females (60%) and 12 males (40%). The mean age of patients with U‐ABL was 32.53 years, comprising 14 females (46.7%) and 16 males (53.3%). For the DFs, the mean age was 22.67 years, with 11 females (73.33%) and 4 males (26.67%).

### 3.2 Immunohistochemistry

**FIGURE 2:**
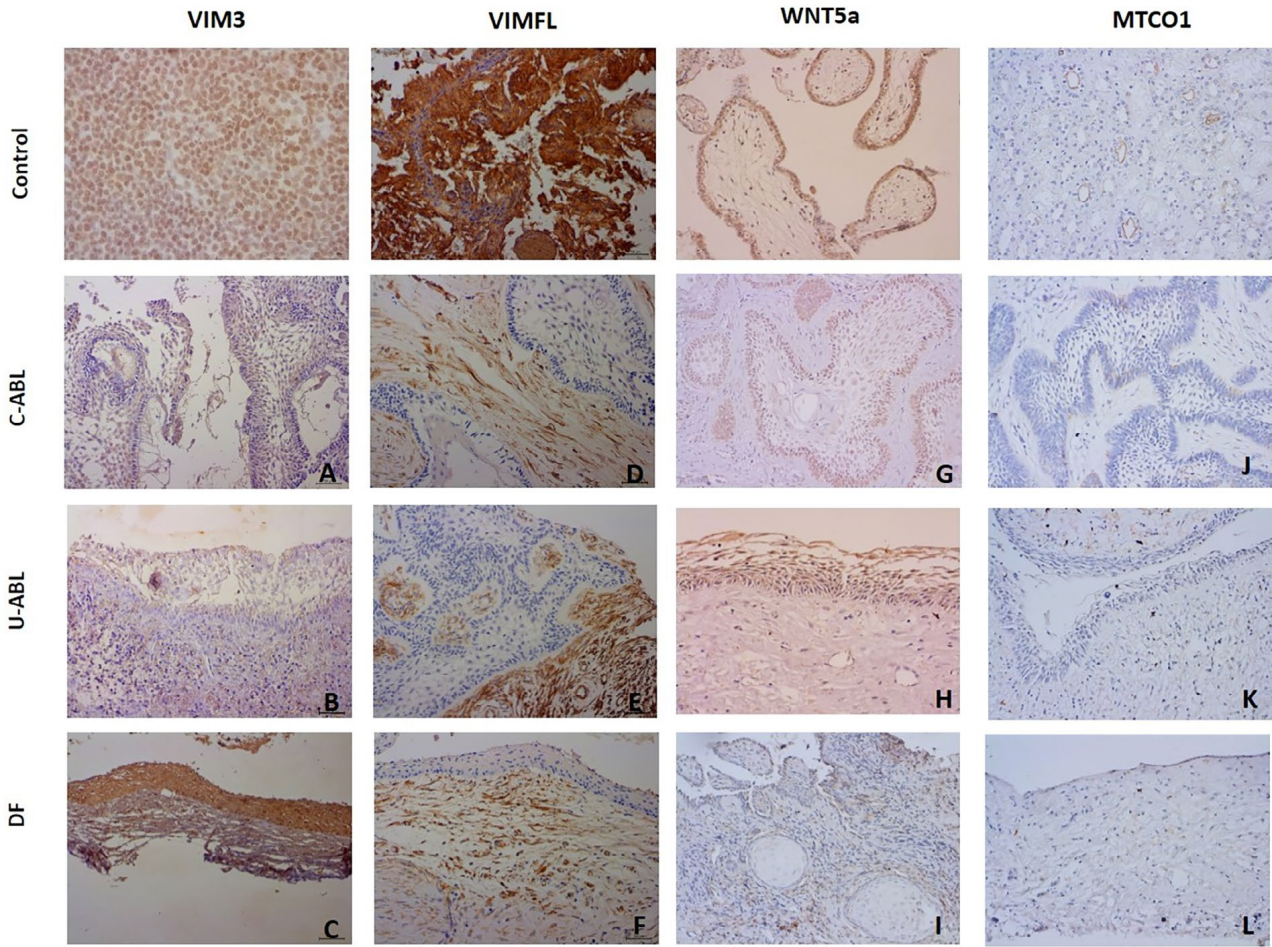
VIM3 positivity in basal and stellate reticulum cells of tumor islands in C‐ABL (A). Basal cells, stellate reticulum cells, and surface cubic cells of the lining epithelium positive with VIM3 in U‐ABL (B). VIM3 positivity in basal cuboidal cells and suprabasal flat cells of the epithelial lining in DF (C). Weak VIM3 staining in stromal connective tissue in C‐ABL and U‐ABL (A, B), but no VIM3 expression was seen in DFs (C). There was strong VIMFL positivity in stromal connective tissue in C‐ABL, U‐ABL, and DF, while VIMFL was not detected in epithelial components (D, E, F). WNT5a positivity in the basal and stellate reticulum cells of C‐ABL and in the basal, stellate reticulum, and superficial cuboidal cells of the laying epithelium in U‐ABL (G, H). The basal cells of the epithelium and inflammatory cells show WNT5a positivity in DF (I). MTCO1 positivity in cells at the periphery of islands in C‐ABL and in basal cells in U‐ABL (J, K). Weak MTCO1 positivity in basal cells of the epithelium in DFs (L). (A‐L, DAB ×200)

In C‐ABL cases, VIM3 positivity was observed in the basal and stellate reticulum cells of the tumor islands. In U‐ABL cases, VIM3 was positively stained in the basal cells of the lining epithelium, stellate reticulum cells, and superficial cuboidal cells. In DFs, VIM3 positivity was noted in the basal cuboidal cells of the lining epithelium and suprabasal squamous cells. While weak VIM3 staining was observed in the connective tissue components of C‐ABL and U‐ABL, VIM3 expression was evident in odontogenic epithelial rests in DFs (Figure 2A–C). Comparison of VIM3 immunoexpression revealed higher expression in C‐ABL cases (*p* < 0.0001). A statistically significant difference was observed in both C‐ABL and U‐ABL compared to DFs (*p* < 0.0001) (Figure 2).

VIMFL staining was notably strong in the connective tissue cells across C‐ABL, U‐ABL, and DF cases. In contrast, no VIMFL positivity was observed in the epithelial components in any of the cases (Figure 2D–F).

**FIGURE 3:**
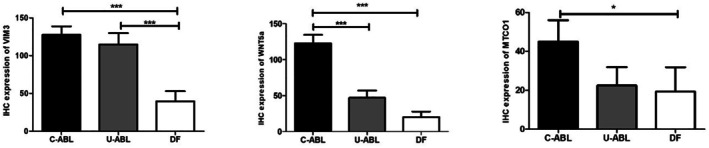
Evaluation of IHC results for VIM3, WNT5a, and MTCO1 between C‐ABL, U‐ABL, and DF (*p < 0.05, **p < 0.01, ***p < 0.001).

WNT5a staining revealed positivity in the basal and stellate reticulum cells of C‐ABLs. In U‐ABLs, WNT5a positivity was noted in the basal, stellate reticulum, and superficial cuboidal cells of the lining epithelium. In DFs, WNT5a positivity was observed in the basal cells of the epithelium and in inflammatory cells within the connective tissue (Figure 2G–I). Comparison of the groups revealed that WNT5a immunoexpression was significantly higher in the C‐ABLs (*p* < 0.0001). Both C‐ABL and U‐ABL showed a statistically significant difference when compared to DFs (*p* < 0.0001) (Figures 2 and 3).

MTCO1 staining showed positivity in the basal cells of C‐ABLs and in the basal cells in U‐ABLs. In DFs, weak staining was observed in the basal cells of the epithelium (Figure 2J–L). Comparison among the groups revealed significantly higher MTCO1 expression in the C‐ABL cases (*p* = 0.0327) (Figures 2 and 3).

### 3.3 qRT‐PCR

**FIGURE 4:**
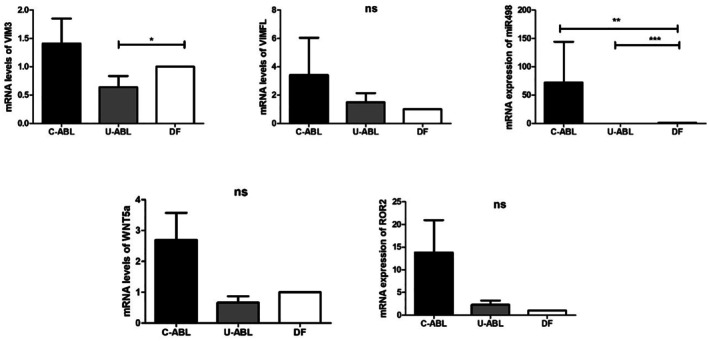
Evaluation of qRT‐PCR results for VIM3, VIMFL, miR498, WNT5a, and ROR2 between C‐ABL, U‐ABL, and DF (*p < 0.05, **p < 0.01, ***p < 0.001).

Evaluation of the qRT‐PCR results revealed a statistically significant difference in VIM3 expression levels between U‐ABL and DF groups in terms of relative mRNA expression (*p* = 0.0384). When comparing miR‐498 levels, a statistically significant difference was found compared to the DF group (*p* < 0.0001). The groups did not show any statistically significant differences in the expression levels of WNT5a, VIMFL, and ROR2 (*p* > 0.05) (Figure 4).

## 4 Discussion

Ameloblastoma (ABL), although known as a benign tumor, holds clinical significance in dentistry due to its locally aggressive nature. ABL stands out as the most common benign epithelial odontogenic tumor affecting the jaw, accounting for approximately 10% of all tumors originating in the mandible and maxilla (Ghai 2022). According to the last classification by WHO in 2022, ABLs are categorized into four subtypes (Soluk‐Tekkesin and Wright 2022). Although these variants originate from the same epithelium, they have different biological behaviors.

It is often difficult for the pathologist to diagnose U‐ABL in incisional biopsy materials without classical diagnostic features and to make the differential diagnosis between C‐ABL and U‐ABLs. In small biopsy materials, solid connective tissue is usually limited and only fragmented epithelial tissue can be found (Ghai 2022). Therefore, we preferred to include those two commons (U‐ABL and C‐ABL) but diagnostically and biologically critical variants in our study. Since VIM3 expression was shown in the small biopsies of prostate carcinomas (Köditz et al. 2021b), it could be a candidate for the differential diagnostic marker for ABL types.

This is the first study to investigate the expression profile of VIM3 in odontogenic tumors with special reference to ABLs. This study showed the immunoexpression profiles of VIM3, WNT5a, VIMFL (clone V9, which specifically targets the tail region of Vimentin), and MTCO1 across C‐ABL, U‐ABL, and DF cases, alongside gene expression differences via qRT‐PCR. The findings highlight significant molecular and cellular differences between the groups, emphasizing key pathways in the tumorigenesis of ABLs.

Vimentin protein is an important component of intermediate filaments and is expressed by normal mesenchymal cells. Vimentin maintains cellular integrity and provides resistance to stress (Paulin et al. 2022). Overexpression of Vimentin has been found in many epithelial tumors, including lung cancer, prostate cancer, gastrointestinal tumors, breast cancer, and central nervous system tumors (Satelli and Li 2011). Although increased Vimentin expression in tumors is associated with tumor invasion and poor prognosis, its exact role in ABL progression remains controversial. VIM3 protein is a truncated variant of Vimentin that exhibits distinct biological functions as a result of its distinctive C‐terminal truncation. Since the discovery of VIM3, it has been studied in many benign and malignant aggressive tumors, including renal cell carcinoma, oncocytoma (von Brandenstein et al. 2021) and prostate cancer (Köditz et al. 2021a, 2021b), but has not been identified in odontogenic tumors. VIM3 is the only histological marker used to differentiate chromophobe eosinophilic variant renal carcinoma and oncocytoma. Oncocytoma is a benign tumor with a high number and activity of mitochondria, unlike chromophobe eosinophilic variant renal carcinoma (von Brandenstein et al. 2021, 2015). The indication that VIM3 could be linked to mitochondrial functions arises from the discovery that this protein is notably overexpressed in oncocytomas, known for its high mitochondrial activity (von Brandenstein et al. 2015). Farman et al. (1986) demonstrated the presence of a substantial number of mitochondria within the epithelial/connective tissue junction of follicular ABLs. A recent study demonstrated that mitochondrial dysregulation and imbalanced mitochondrial dynamics were observed in ABL among the odontogenic lesions (Suppamaeteekulwat et al. 2021). In our study, in ABLs, the VIMFL levels are reduced, while the expression of VIM3, a truncated vimentin isoform, is upregulated. Taken all together, VIM3 may play a role in the tumorigenesis of ABLs via affecting the mitochondrial functioning.

The elevated expression of VIM3 in C‐ABL, as evidenced by both immunohistochemistry and qRT‐PCR, further supports the involvement of Vimentin isoforms in ABL pathogenesis. The significant difference in VIM3 expression between U‐ABL and DF (*p* = 0.0384) highlights a potential biomarker for distinguishing between these lesions, aligning with (Qiao et al. 2020) who demonstrated that cytoskeletal dynamics play a crucial role in ameloblastoma cell migration. This suggests that VIM3, through its influence on cytoskeletal organization, may contribute to the invasive nature of ABLs.

On the other hand, our study shows significant differences in the expression of VIM3 and miR‐498 between U‐ABL, C‐ABL, and DF groups, shedding light on potential mechanisms involved in ABL pathogenesis. The significantly higher expression of VIM3 in U‐ABL compared to DFs (*p* = 0.0384), along with the increased levels of miR‐498 in U‐ABL (*p* < 0.0001), suggests that miR‐498 plays a pivotal role in driving VIM3 production in these tumors. Already, previous studies have shown that miR‐498 can induce protein truncation, leading to the formation of VIM3 from VIMFL (von Brandenstein et al. 2018, 2015). In renal cell carcinoma, VIM3 has been associated with more aggressive phenotypes, indicating its potential role as a marker for malignancy (Köditz et al. 2021b). Similarly, the increased VIM3 expression in ABLs suggests that VIM3 could contribute to the invasive nature of these tumors, particularly through its role in cytoskeletal dynamics and cellular migration.

The noted increase of miR‐498 in U‐ABL compared to DF (*p* < 0.0001) further supports the involvement of microRNA‐mediated regulation in ABL biology. This aligns with the findings of von Brandenstein et al. (2018), who proposed that microRNAs, such as miR‐498, might significantly influence protein expression and tumor dynamics via post‐transcriptional modifications, including protein truncation. The significant increase in miR‐498 levels indicates that miR‐498 could function as a biomarker for differentiating benign from more aggressive odontogenic tumors. These findings not only deepen our understanding of the molecular mechanisms underlying ABL pathogenesis but also point to miR‐498 and VIM3 as promising targets for future diagnostic and therapeutic approaches in the management of odontogenic tumors.

Moreover, the lack of VIMFL positivity in epithelial components in any of the cases, contrasted with strong staining in connective tissue cells, suggests a complex interplay between the stromal environment and tumor cells. VIM3 may retain different functional properties compared to VIMFL, influencing tumor–stroma interactions in a unique way. The result correlates with the study that identified specific interactions at the epithelium–connective tissue junction in ABLs, suggesting that stromal cells may play a role in tumor development (Farman et al. 1986). This stromal involvement is consistent with the fact that Vimentin is a key player in epithelial‐mesenchymal transition (EMT) (Usman et al. 2021), and its truncated form, VIM3, could similarly alter cellular adhesion and migration dynamics, further contributing to ABL invasion potential.

WNT5a immunoexpression was notably higher in C‐ABL cases (*p* < 0.0001), with significant differences observed in both C‐ABL and U‐ABL compared to DF. This underscores the role of the WNT pathway in ABLs, particularly in C‐ABLs. Suppamaeteekulwat et al. (2021) reported that the modulation of mitochondrial and cytoskeletal dynamics can affect ABL cell migration through the WNT5a/Ca + signaling pathway. Qiao et al. (2020) have shown that WNT5a is involved in non‐canonical WNT signaling, influencing cell migration and mitochondrial dynamics. In this context, the higher WNT5a levels observed in C‐ABL cases may indicate a more aggressive phenotype. The differential localization of WNT5a in basal and stellate reticulum cells in C‐ABL cases, and in basal and cuboidal cells in U‐ABLs, further supports its role in diverse cellular processes across different ABL subtypes.

The increased MTCO1 expression in C‐ABL (*p* = 0.0327) and its specific localization to basal cells suggests a mitochondrial contribution to the metabolic demands of the tumor, particularly in C‐ABL. The mitochondrial involvement, particularly in basal cells, may be linked to the higher proliferative and invasive potential observed in C‐ABL. This is thought to be explained by studies suggesting that mitochondrial function and dynamics are altered in odontogenic tumors (Suppamaeteekulwat et al. 2021).

Interestingly, despite the significant immunohistochemical findings for WNT5a and VIMFL, no statistically significant differences were observed at the mRNA level between groups. This discrepancy between protein and gene expression levels suggests post‐transcriptional regulation or protein stability as factors influencing the observed immunoexpression patterns. The lack of significant differences in ROR2 expression, despite its known role as a WNT5a receptor (Bo et al. 2013), further highlights the complexity of tumorigenesis in ABLs and suggests that additional factors such as post‐transcriptional modifications may regulate WNT activity in these tumors.

In conclusion, the differential expression of VIM3, WNT5a, and MTCO1, alongside miR‐498 levels, underscores the complex molecular landscape of ABLs. The significant differences in VIM3 and miR‐498 expression between U‐ABL, C‐ABL, and DF cases highlight the potential role of these molecules in ABL progression. miR‐498 appears to regulate the truncation of Vimentin into VIM3, which in turn may promote tumor invasiveness through alterations in cytoskeletal organization. Further studies, particularly focusing on post‐transcriptional mechanisms and mitochondrial dynamics, are warranted to better understand the biological underpinnings of these tumors. It is believed that understanding the specific expression profile of VIM3 in the context of ABL will pave the way for advanced studies aimed at comprehending the complex nature of this tumor. Our results represent a significant step forward, representing the first study to elucidate VIM3 expression in odontogenic tumors, focusing on ABL.

## Author Contributions

**Sibel Elif Gultekin:** conceptualization, investigation, writing – original draft, methodology, validation, writing – review and editing, supervision, data curation, project administration. **Melanie von Brandenstein:** conceptualization, investigation, writing – original draft, methodology, validation, writing – review and editing, data curation, supervision, project administration. **Emre Baris:** investigation, writing – original draft, methodology, validation. **Ipek Atak Secen:** investigation, writing – original draft, methodology, validation, visualization. **Leyla Arslan Bozdag:** investigation, writing – original draft, methodology, validation, visualization. **Heike Goebel:** investigation, writing – original draft, methodology, validation.

## Conflicts of Interest

The authors declare no conflicts of interest.

## Data Availability

The data that support the findings of this study are available from the corresponding author upon reasonable request.

## References

[odi15396-bib-0001] Anastas, J. N. , and R. T. Moon . 2013. “WNT Signalling Pathways as Therapeutic Targets in Cancer.” Nature Reviews Cancer 13, no. 1: 11–26. 23258168 10.1038/nrc3419

[odi15396-bib-0002] Avilés‐Salas, A. , S. Muñiz‐Hernández , H. A. Maldonado‐Martínez , et al. 2017. “Reproducibility of the EGFR Immunohistochemistry Scores for Tumor Samples From Patients With Advanced Non‐Small Cell Lung Cancer.” Oncology Letters 13, no. 2: 912–920. 28356978 10.3892/ol.2016.5512 PMC5351342

[odi15396-bib-0003] Barış, E. , B. Sengüven , S. Bozkaya , and S. S. Ergüven . 2015. “Unicystic Ameloblablastoma With Diverse Mural Proliferation: A Case Report.” Oral Surgery, Oral Medicine, Oral Pathology and Oral Radiology 119, no. 3: e152.

[odi15396-bib-0004] Barnes, L. 2005. Pathology and Genetics of Head and Neck Tumours. IARC.

[odi15396-bib-0005] Bo, H. , S. Zhang , L. Gao , et al. 2013. “Upregulation of Wnt5a Promotes Epithelial‐To‐Mesenchymal Transition and Metastasis of Pancreatic Cancer Cells.” BMC Cancer 13: 496. 10.1186/1471-2407-13-496. 24156409 PMC4077028

[odi15396-bib-0006] Effiom, O. , O. Ogundana , A. Akinshipo , and S. Akintoye . 2018. “Ameloblastoma: Current Etiopathological Concepts and Management.” Oral Diseases 24, no. 3: 307–316. 28142213 10.1111/odi.12646

[odi15396-bib-0007] Eskuri, M. , N. Kemi , and J. H. Kauppila . 2021. “Monocarboxylate Transporters 1 and 4 and MTCO1 in Gastric Cancer.” Cancers 13, no. 9: 2142. 33946786 10.3390/cancers13092142 PMC8124264

[odi15396-bib-0008] Farman, A. G. , A. R. Gould , and E. Merrell . 1986. “Epithelium‐Connective Tissue Junction in Follicular Ameloblastoma and Ameloblastic Fibroma: An Ultrastructural Analysis.” International Journal of Oral and Maxillofacial Surgery 15, no. 2: 176–186. 10.1016/s0300-9785(86)80138-7. 3083021

[odi15396-bib-0009] Ghai, S. 2022. “Ameloblastoma: An Updated Narrative Review of an Enigmatic Tumor.” Cureus 14, no. 8: e27734. 10.7759/cureus.27734. 36127985 PMC9481193

[odi15396-bib-0010] Köditz, B. , A. Stog , H. Göbel , et al. 2021b. “A New Prostate Cancer Surrogate Marker‐Vimentin 3?” Journal of Oncology Medicine and Practice 4, no. 3: 402–408.

[odi15396-bib-0011] Köditz, B. , A. Stog , H. Göbel , et al. 2021a. “Vimentin 3 Expression in Prostate Cancer Cells.” Anticancer Research 41, no. 1: 169–174. 10.21873/anticanres.14762. 33419810

[odi15396-bib-0012] Kumawat, K. , and R. Gosens . 2016. “WNT‐5A: Signaling and Functions in Health and Disease.” Cellular and Molecular Life Sciences 73: 567–587. 26514730 10.1007/s00018-015-2076-y PMC4713724

[odi15396-bib-0013] Lin, C.‐S. , H.‐T. Lee , M.‐H. Lee , et al. 2016. “Role of Mitochondrial DNA Copy Number Alteration in Human Renal Cell Carcinoma.” International Journal of Molecular Sciences 17, no. 6: 814. 27231905 10.3390/ijms17060814 PMC4926348

[odi15396-bib-0014] Mendenhall, W. M. , J. W. Werning , R. Fernandes , R. S. Malyapa , and N. P. Mendenhall . 2007. “Ameloblastoma.” American Journal of Clinical Oncology 30, no. 6: 645–648. 18091060 10.1097/COC.0b013e3181573e59

[odi15396-bib-0015] Paulin, D. , A. Lilienbaum , S. Kardjian , O. Agbulut , and Z. Li . 2022. “Vimentin: Regulation and Pathogenesis.” Biochimie 197: 96–112. 10.1016/j.biochi.2022.02.003. 35151830

[odi15396-bib-0016] Prgomet, Z. , T. Andersson , and P. Lindberg . 2017. “Higher Expression of WNT 5A Protein in Oral Squamous Cell Carcinoma Compared With Dysplasia and Oral Mucosa With a Normal Appearance.” European Journal of Oral Sciences 125, no. 4: 237–246. 28603941 10.1111/eos.12352 PMC5519933

[odi15396-bib-0017] Prgomet, Z. , L. Axelsson , P. Lindberg , and T. Andersson . 2015. “Migration and Invasion of Oral Squamous Carcinoma Cells Is Promoted by WNT 5A, a Regulator of Cancer Progression.” Journal of Oral Pathology & Medicine 44, no. 10: 776–784. 25459554 10.1111/jop.12292

[odi15396-bib-0018] Qiao, X. , X. Niu , J. Shi , et al. 2020. “Wnt5a Regulates Ameloblastoma Cell Migration by Modulating Mitochondrial and Cytoskeletal Dynamics.” Journal of Cancer 11, no. 18: 5490–5502. 32742496 10.7150/jca.46547 PMC7391189

[odi15396-bib-0019] Satelli, A. , and S. Li . 2011. “Vimentin in Cancer and Its Potential as a Molecular Target for Cancer Therapy.” Cellular and Molecular Life Sciences 68: 3033–3046. 21637948 10.1007/s00018-011-0735-1 PMC3162105

[odi15396-bib-0020] Soluk‐Tekkesin, M. , and J. M. Wright . 2022. “The World Health Organization Classification of Odontogenic Lesions: A Summary of the Changes of the 2022 (5th) Edition.” Turkish Journal of Pathology 38, no. 2: 168–184. 35578902 10.5146/tjpath.2022.01573 PMC9999699

[odi15396-bib-0021] Sukarawan, W. , D. Simmons , C. Suggs , K. Long , and J. T. Wright . 2010. “WNT5A Expression in Ameloblastoma and Its Roles in Regulating Enamel Epithelium Tumorigenic Behaviors.” American Journal of Pathology 176, no. 1: 461–471. 20008136 10.2353/ajpath.2010.090478 PMC2797904

[odi15396-bib-0022] Suppamaeteekulwat, B. , N. Apaijai , Y. Aschaitrakool , et al. 2021. “The Differences in Mitochondrial Function, Mitochondrial Dynamics, and Cell Death Between Odontogenic Cysts/Tumors and Normal Dental Follicles.” Mitochondrion 59: 175–183. 34091078 10.1016/j.mito.2021.06.004

[odi15396-bib-0023] Usman, S. , N. H. Waseem , T. K. N. Nguyen , et al. 2021. “Vimentin Is at the Heart of Epithelial Mesenchymal Transition (EMT) Mediated Metastasis.” Cancers (Basel) 13, no. 19: 4985. 10.3390/cancers13194985. 34638469 PMC8507690

[odi15396-bib-0024] von Brandenstein, M. , S. H. Bernhart , A. Pansky , et al. 2018. “Beyond the 3′UTR Binding‐microRNA‐Induced Protein Truncation via DNA Binding.” Oncotarget 9, no. 32867: 32855. 10.18632/oncotarget.26023. 30214689 PMC6132356

[odi15396-bib-0025] von Brandenstein, M. , J. Herden , B. Köditz , et al. 2021. “Non‐Invasive Urine Markers for the Differentiation Between RCCs and Oncocytoma.” Journal of Clinical Laboratory Analysis 35, no. 5: e23762. 10.1002/jcla.23762. 33960011 PMC8128285

[odi15396-bib-0026] von Brandenstein, M. , K. Puetz , M. Schlosser , et al. 2015. “Vimentin 3, the New Hope, Differentiating RCC Versus Oncocytoma.” Disease Markers 2015: 368534. 10.1155/2015/368534. 25944973 PMC4405285

[odi15396-bib-0027] Wallace, D. C. , and W. Fan . 2009. “The Pathophysiology of Mitochondrial Disease as Modeled in the Mouse.” Genes & Development 23, no. 15: 1714–1736. 19651984 10.1101/gad.1784909 PMC2720256

